# Association between the Pro12Ala Polymorphism of Peroxisome Proliferator-Activated Receptor Gamma 2 and Inflammatory Bowel Disease: A Meta-Analysis

**DOI:** 10.1371/journal.pone.0030551

**Published:** 2012-01-19

**Authors:** Zhi-Feng Zhang, Ning Yang, Gang Zhao, Lei Zhu, Li-Xia Wang

**Affiliations:** 1 Department of Gastroenterology, The First Affiliated Hospital of Dalian Medical University, Dalian, China; 2 Department of Nephrology, The First Affiliated Hospital of Dalian Medical University, Dalian, China; Karolinska Institutet, Sweden

## Abstract

**Background:**

Peroxisome proliferator-activated receptor gamma (PPARγ), a nuclear receptor, has been implicated playing a role in the development of inflammatory bowel disease (IBD). However, previous studies evaluating the association between the PPARγ2 Pro12Ala polymorphism and IBD are inconsistent. We performed a meta-analysis to determine whether the PPARγ2 Pro12Ala mutation was associated with the presence of IBD.

**Methods and Findings:**

Electronic databases were searched for case-control studies evaluating the association between the Pro12Ala mutation and the presence of IBD. Effects were summarized with the methods recommended by the Cochrane Collaboration. A total of 7 studies including 1002 ulcerative colitis (UC) cases, 1090 Crohǹs disease (CD) cases and 1983 controls were involved in this meta-analysis. In the overall analysis, no significant association of this polymorphism with UC or CD was found. In the subgroup analyses in different populations, AlaAla genotype seemed to protect the European Caucasian population against the development of CD (Pro vs Ala: OR = 1.135, 95%CI = 0.951–1.354, *P* = 0.162, Bon = 1.000; ProPro vs ProAla: OR = 1.042, 95%CI = 0.852–1.273, *P* = 0.690, Bon = 1.000; ProPro vs AlaAla: OR = 2.379, 95%CI = 1.110–5.100, *P* = 0.026, Bon = 0.156; ProAla vs AlaAla: OR = 2.315, 95%CI = 1.064–5.037, *P* = 0.034, Bon = 0.204; Pro homozygotes vs Ala positives: OR = 1.094, 95%CI = 0.899–1.330, *P* = 0.371, Bon = 1.000; Pro positives vs Ala homozygotes: OR = 2.360, 95%CI = 1.103–5.053, *P* = 0.027, Bon = 0.162; heterozygotes vs all homozygotes: OR = 0.976, 95%CI = 0.799–1.192, *P* = 0.809, Bon = 1.000). There was no significant association of this polymorphism with UC or CD in the East Asian population and the Turkish population.

**Conclusion:**

AlaAla genotype may be a protective factor in the European Caucasian population against the development of CD in a recessive way.

## Introduction

Inflammatory bowel disease (IBD) clinically classified into ulcerative colitis (UC) and Crohǹs disease (CD) is a non specific chronic intestinal inflammatory disorder with the characteristics of relapses and remissions. When diagnosed, patients with UC or CD need lifelong medications. Moreover, strictures, abscesses, fistulas, extra intestinal involvements and even carcinomas would complicate IBD and eventually affect the progression of IBD. In the last three decades, we have witnessed a time trend increase of the prevalence of IBD especially of CD both in developed and developing regions [1].

Although the treatment of IBD has advanced considerably and guidelines have just been revised, IBD is still incurable [2]. Clarifying the etiology of IBD may help us to find optimal therapies. To date, it is believed that the inappropriate response of the intestinal mucosal immune system to indigenous intestinal flora and other antigens is crucial in IBD development [3]. The mechanism of the inappropriate response is different in UC and CD. The deficiency of the innate immune system which permits intestinal pathogens to break through the intestinal barrier and activate the adaptive immune system may be responsible for the uncontrolled inflammation in CD, while UC is probably caused by the primarily upregulated response of the intestinal mucosal immune system [4].

Peroxisome proliferator-activated receptor gamma (PPARγ), a nuclear receptor, has been implicated as playing some role in the development of UC and CD [5], [6]. One experimental study even showed the beneficial effect of a PPARγ agonist on UC [7]. The CCA-to-GCA (Pro-to-Ala) mutation in codon 12 of exon B of the PPARγ2 is a common single nucleotide polymorphism (SNP) [8]. The Pro12Ala mutation may affect the activity of PPARγ in epithelial cells and immunocytes, and consequently interfere with the susceptibility of a host to develop IBD. In order to elucidate the association between the Pro12Ala mutation and the development of IBD, some molecular epidemiological researches were conducted worldwide. One case-control study conducted in Japan indicated that the Pro12Ala mutation was more frequently found in UC patients [9]. Another study showed a significant association between this mutation and the development of CD [10]. However, a research conducted in China and Holland failed to confirm the association between the Pro12Ala polymorphism and IBD [11]. One reason of the inconsistent results of previous studies may be the sample size not large enough in some studies. Meta-analysis is a well-established method combining all available published data to increase the statistical power of the analysis. Hence we performed this meta-analysis to determine whether the Pro12Ala mutation was associated with the development of IBD.

## Methods

### Searching strategies

PubMed (1966 to June 2011) and Scopus (1966 to June 2011) were searched using the combinations of text words and medical subject headings (MeSH) including “inflammatory bowel disease”, “ulcerative colitis”, “Crohn's disease”, “IBD”, “UC”, “CD”, “peroxisome proliferator-activated receptor”, “PPAR”, “polymorphism”, “polymorphisms”, “single nucleotide”, “allele” and “genotype”. Chinese medical databases including Wanfang database (1982 to June 2011), China National Knowledge Infrastructure (CNKI, 1994 to June 2011) and Chinese Biomedical Literature Database (CBM, 1978 to June 2011) were also searched for relevant articles. We searched reference lists, relevant meta-analyses and reviews in order to get additional articles.

### Study selection criteria

The following selection criteria were employed in this meta-analysis: 1) Case-control studies evaluating the association between the PPARγ2 Pro12Ala mutation and the presence of IBD. 2) Diagnosis of IBD according to clinical manifestations, radiological changes, endoscopic manifestations, and histological evaluations comprehensively. 3) Hardy-Weinberg equilibrium fulfilled in the control arm of each research. 4) Articles with a full text not just an abstract. 5) Articles providing raw data or Odds ratio (OR) and its 95% confidence interval (CI) for each comparison. Exclusion criteria were as the follows: 1) Repetitive publications. 2) Family based case-control studies.

### Data extraction

Two reviewers screened the titles and abstracts of potentially relevant articles. And the full texts of highly relevant articles would be thoroughly read by two reviewers. When a discrepancy was encountered, a third reviewer would be referred to and the decision would be made through discussions. Publication year, region the study was conducted in, ethnicity, available allele and genotype frequencies in each arm and the method of polymorphism assessment of each study were exacted.

### Assessment of the risks of bias

We also assessed the potential risks of bias with the items including 1) Selection bias (differential selections of cases and controls; selections based on UC or CD severity), 2) Information bias (quality control measures in the genotyping process, blinding of laboratory personnel or researchers and phenotype misclassification) and 3) confounding factors (cases and controls matching).

### Data analysis

Hardy-Weinberg equilibrium was assessed using the χ2 test. Paired combinations of genotypes were employed to determine the hereditary models: 1) an allelic analysis (Pro vs Ala); 2) a genotypic analysis (ProPro vs AlaAla, ProPro vs ProAla, ProAla vs AlaAla) and 3) another genotypic analysis comparing each genotype with the other two (Pro homozygotes vs Ala positives, Pro positives vs Ala homozygotes, heterozygotes vs all homozygotes). If the comparisons of ProPro vs AlaAla, ProPro vs ProAla and Pro homozygotes vs Ala positives were statistically significant, the Ala allele would be a dominant allele. If the comparisons of ProPro vs AlaAla, ProAla vs AlaAla and Pro positives vs Ala homozygotes were statistically significant, the Ala allele would be a recessive allele. OR and its 95% CI were calculated with the methods recommended by the Cochrane Collaboration, and a fixed value of 0.5 was added to all cells of study results tables where no events were observed in one or both groups [12]. The Cochrane Q χ2 test and the CI overlapping status of each selected study were used to detect the heterogeneity among studies. The *I^2^* statistics was also used to evaluate the risks of heterogeneity among studies: 0%–40% represented no risk of heterogeneity, 30%–60% represented a low risk of heterogeneity, 50%–90% represented substantial heterogeneity and 75%–100% represented considerable heterogeneity [12]. If the *P* value was more than 0.1, the *I^2^* statistics indicating no or a low risk of heterogeneity and the CI of each study overlapped, a fixed model was employed and the Mantel-Haenszel method was implemented to synthesize data. If the *P* value was less than 0.1, the *I^2^* statistics indicating substantial or considerable heterogeneity or the CI of each study did not overlapped, a random model was employed and the DerSimonian-Laird method was applied to synthesize data. Funnel plots were used to examine bias in the results of the meta-analyses [13], [14]. The step down Bonferroni method was used for the multiple comparison adjustments [15]. Stata 11.0 software (StataCorp LP, College Station, Texas, USA) was used for meta-analyses and Hardy-Weinberg equilibrium tests. R 2.13.0 software (The R Foundation for Statistical Computing, http://cran.csdb.cn/) was used for the step down Bonferroni adjustments (Bon). Values of *P*<0.05 were considered statistically significant. All the above methods had been used for pooling data in two previous published genetic association meta-analyses [16.17].

## Results

### Characteristics of selected studies

**Figure 1:**
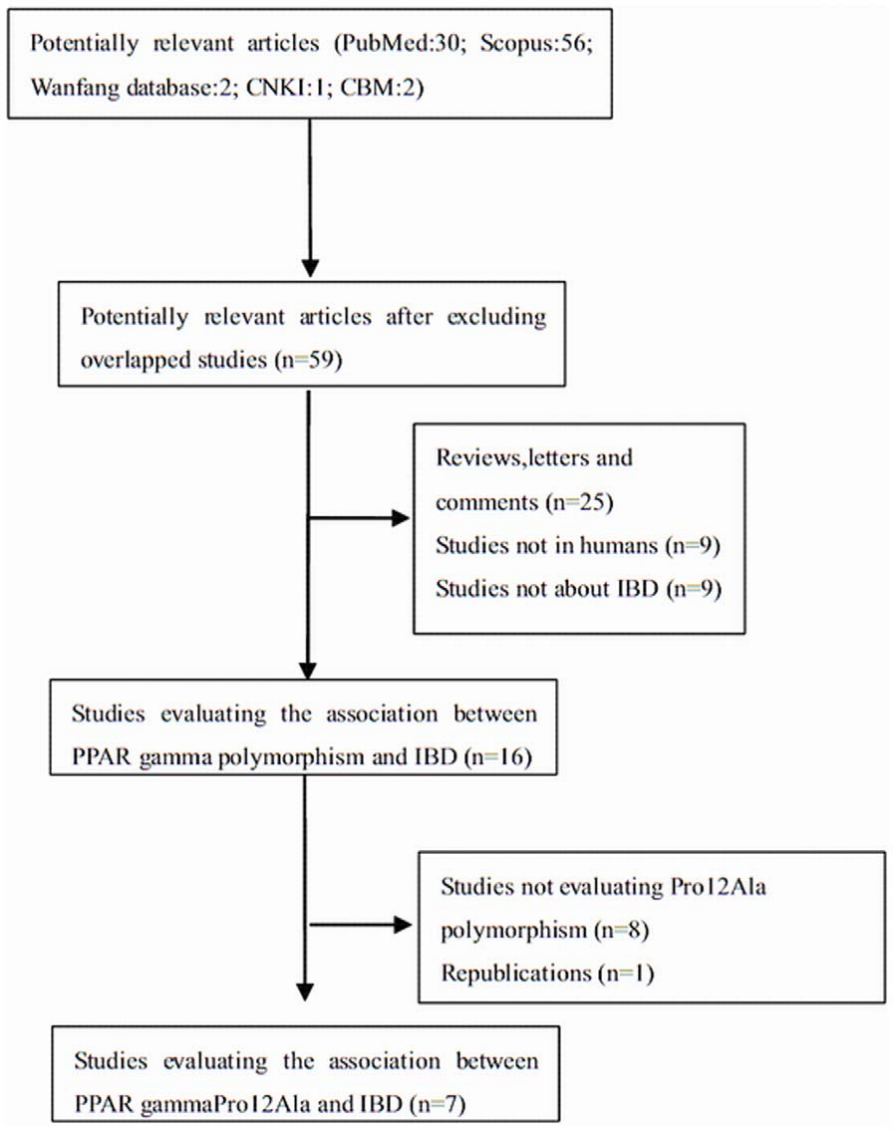
Flow diagram of the study selection process.

91 articles were identified during premature searches with our searching strategy of the five databases (PubMed: 30; Scopus: 56; Wanfang database: 2; CNKI: 1; CBM: 2). 59 articles were retrieved after excluding overlapping studies. After excluding reviews, animal studies, comments, letters and studies not evaluating IBD, 16 studies assessing the association between the PPARγ polymorphism and IBD were found. Among the remaining 16 articles, 8 studies did not evaluate the Pro12Ala mutation and one republication was identified. Only 7 articles met our selection criteria. After searching reference lists, relevant meta-analyses and reviews, we did not find additional studies. The selection process is illustrated in Figure 1. These 7 studies including 1002 UC cases, 1090 CD cases and 1983 controls were involved in this meta-analysis [9]–[11], [18]–[21]. Among the selected researches, 3 studies were conducted in the European Caucasian population, 3 studies were conducted in the East Asian population and one study was conducted in the Turkish population. The control arm of each study conformed to the Hardy-Weinberg equilibrium. The characteristics of selected studies are illustrated in Table 1 and Table 2. The PRISMA Checklist is shown in Text S1. The detailed searching process of Scopus is demonstrated in Text S2.

**Table 1:** Characteristics of included studies evaluating the association between the PPARγ Pro12Ala mutation and the presence of IBD.

Author	Year	Location	Ethnicity	Case and control selection	SNP method
Atug O [18]	2008	Turkey	Turkish	Case: patients diagnosed in medical institutions	MS-PCR
				Control: healthy volunteers matched for age and gender	
Wang F [19]	2008	Japan	East Asian	Case: patients diagnosed in medical institutions	PCR-RFLP
				Control: healthy control subjects without detailed descriptions of matching methods	
Ferreira P [20]	2010	Portugal	European Caucasian	Case: patients diagnosed in medical institutions	PCR-RFLP
				Control: healthy blood donors without detailed descriptions of matching methods	
Shrestha UK [11]	2010	China	East Asian	Case: patients diagnosed in medical institutions	PCR-RFLP
				Control: healthy controls matched for age and gender	
Andersen V [21]	2011	Denmark	European Caucasian	Case: patients diagnosed in medical institutions	Taqman
				Control: healthy blood donors without detailed descriptions of matching methods	
Aoyagi Y [9]	2010	Japan	East Asia	Case: patients diagnosed in medical institutions	RT-PCR
				Control: healthy controls matched for age and gender	
Poliska S [10]	2011	Hungary	European Caucasian	Case: patients diagnosed in medical institutions	Taqman
				Control: healthy controls matched for age and gender	

SNP: single nucleotide polymorphism; MS-PCR: mutagenically separated polymerase chain reaction; PCR-RFLP: polymerase chain reaction-restriction fragment length polymorphism; Taqman: Taqman SNP genotyping assays; RT-PCR: real time polymerase chain reaction.

**Table 2:** Allele and genotype frequencies of the arms of included studies evaluating the association between the PPARγ Pro12Ala mutation and the presence of IBD.

Author	Arms	Pro	Ala	Pro/Pro	Pro/Ala	Ala/Ala	HWE test
Atug O [18]	UC (n = 45)	83	7	38	7	0	
	CD (n = 69)	127	11	58	11	0	
	Control (n = 100)	187	13	87	13	0	*P* = 0.487
Wang F [19]	UC (n = 118)	231	5	113	5	0	
	Control (n = 142)	277	7	135	7	0	*P* = 0.763
Ferreira P [20]	CD (n = 90)	163	17	74	15	1	
	Control (n = 116)	209	23	95	19	2	*P* = 0.371
Shrestha UK [11]	UC (n = 212)	400	24	189	22	1	
	CD (n = 32)	61	3	29	3	0	
	Control(n = 220)	416	24	198	20	2	*P* = 0.079
Andersen V [21]	UC (n = 495)	844	146	364	116	15	
	CD (n = 327)	564	90	240	84	3	
	Control (n = 779)	1315	243	549	217	13	*P* = 0.105
Aoyagi Y [9]	UC (n = 29)	52	6	25	2	2	
	CD (n = 10)	20	0	10	0	0	
	Control (n = 134)	264	4	130	4	0	*P* = 0.861
Poliska S [10]	UC (n = 103)	178	28	77	24	2	
	CD (n = 562)	990	134	433	124	5	
	Control (n = 492)	854	130	375	104	13	*P* = 0.083

UC: ulcerative colitis; CD: Crohn's disease; HWE test: Hardy-Weinberg equilibrium test.

### Assessment of the risks of bias

Selection bias: Cases were selected from diagnosed UC and CD patients in medical institutions in all the included studies. 3 studies recruited healthy blood donors as controls [10], [20], [21]. One study recruited healthy medical staff as controls [11]. While the other 3 studies only described healthy subjects as controls. Only one study used colonoscopy to exclude IBD in the controls [9]. Only one study described the severity of cases, which was mild to moderate [20].

Information bias: 3 studies used polymerase chain reaction-restriction fragment length polymorphism (PCR-RFLP) as the single nucleotide polymorphism (SNP) detection method [11], [19], [20]. Mutagenically separated polymerase chain reaction (MS-PCR) was employed by one study to detect SNP [18]. Two studies used Taqman SNP genotyping assays (Taqman) to detect SNP [10], [21]. One study detected SNP with real time polymerase chain reaction (RT-PCR) [9]. Investigators were blinded to data in two studies [19], [21]. Negative and positive controls were processed with each batch of samples and all experiments were repeated twice to ensure consistency for quality control purposes in one study [11]. One study genotyped 10% of the samples again to confirm reproducibility [21]. No phenotype misclassification was reported in the selected studies.

Confounding factors: Age and gender distribution were comparable among arms in most studies, while controls were slightly older as compared to CD patients in one study [20]. Regarding the possible influence of the Pro12Ala polymorphism in PPARγ2 gene on the risk of development of the type 2 diabetes mellitus, one study excluded diabetics from the study and control groups [18].

### Meta-analysis

**Figure 2:**
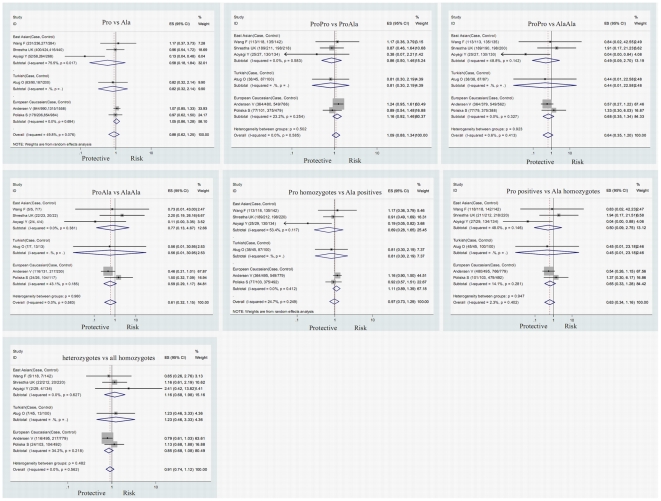
Forest plots for meta-analyses of the association between the PPARγ Pro12Ala mutation and the presence of UC.

**Figure 3:**
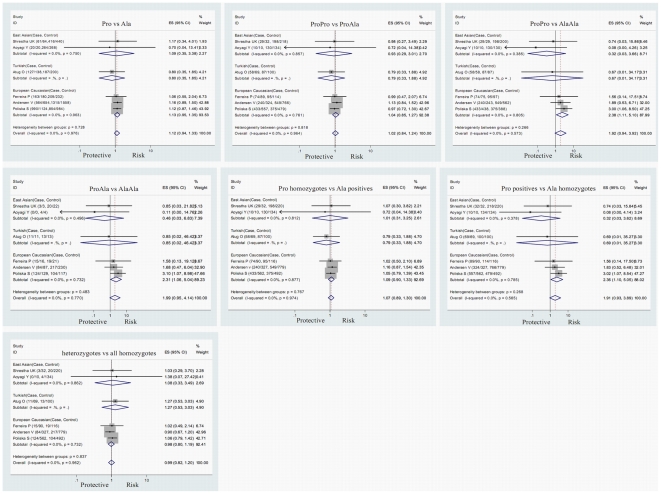
Forest plots for meta-analyses of the association between the PPARγ Pro12Ala mutation and the presence of CD.

The CI overlapping status, Cochrane Q χ2 tests, *I^2^* statistics and the detailed meta-analysis results are shown in Figure 2 and Figure 3.

### Meta-analysis in the overall population

The association between the PPARγ Pro12Ala polymorphism and UC was investigated in 6 studies with 1002 UC cases and 1867 controls [9]–[11], [18], [19], [21]. There was no significant association between this polymorphism and UC in the overall population (Pro vs Ala: OR = 0.884, 95%CI = 0.625–1.251, *P* = 0.486, Bon = 1.000; ProPro vs ProAla: OR = 1.088, 95%CI = 0.883–1.340, *P* = 0.429, Bon = 1.000; ProPro vs AlaAla: OR = 0.644, 95%CI = 0.347–1.197, *P* = 0.165, Bon = 1.000; ProAla vs AlaAla: OR = 0.606, 95%CI = 0.320–1.149, *P* = 0.125, Bon = 0.875; Pro homozygotes vs Ala positives: OR = 0.968, 95%CI = 0.727–1.289, *P* = 0.823, Bon = 1.000; Pro positives vs Ala homozygotes: OR = 0.625, 95%CI = 0.337–1.159, *P* = 0.136, Bon = 0.952; heterozygotes vs all homozygotes: OR = 0.910, 95%CI = 0.739–1.120, *P* = 0.372, Bon = 1.000). Between-study heterogeneity was detected in the contrasts of Pro vs Ala and Pro homozygotes vs Ala positives. The association between the PPARγ Pro12Ala polymorphism and CD was investigated in 6 studies with 1090 CD cases and 1841 controls [9]–[11], [18], [20], [21]. No significant association between this polymorphism and CD in the overall population was found either (Pro vs Ala: OR = 1.117, 95%CI = 0.941–1.325, *P* = 0.204, Bon = 1.000; ProPro vs ProAla: OR = 1.024, 95%CI = 0.845–1.242, *P* = 0.807, Bon = 1.000; ProPro vs AlaAla: OR = 1.918, 95%CI = 0.938–3.922, *P* = 0.074, Bon = 0.518; ProAla vs AlaAla: OR = 1.986, 95%CI = 0.953–4.139, *P* = 0.067, Bon = 0.469; Pro homozygotes vs Ala positives: OR = 1.075, 95%CI = 0.890–1.297, *P* = 0.454, Bon = 1.000; Pro positives vs Ala homozygotes: OR = 1.906, 95%CI = 0.933–3.893, *P* = 0.077, Bon = 0.539; heterozygotes vs all homozygotes: OR = 0.991, 95%CI = 0.818–1.201, *P* = 0.927, Bon = 1.000). Between-study heterogeneity was not detected in all the contrasts.

### Analysis in the European Caucasian population

The association between the PPARγ Pro12Ala polymorphism and UC in the European Caucasian population was investigated in 2 studies with 598 UC cases and 1271 controls [10], [21]. There was no significant association between the Pro12Ala polymorphism and UC in the European Caucasian population (Pro vs Ala: OR = 1.047, 95%CI = 0.858–1.277, *P* = 0.651, Bon = 1.000; ProPro vs ProAla: OR = 1.157, 95%CI = 0.917–1.459, *P* = 0.219, Bon = 1.000; ProPro vs AlaAla: OR = 0.680, 95%CI = 0.346–1.335, *P* = 0.263, Bon = 1.000; ProAla vs AlaAla: OR = 0.586, 95%CI = 0.293–1.173, *P* = 0.131, Bon = 0.917; Pro homozygotes vs Ala positives: OR = 1.109, 95%CI = 0.886–1.388, *P* = 0.365, Bon = 1.000; Pro positives vs Ala homozygotes: OR = 0.653, 95%CI = 0.334–1.280, *P* = 0.215, Bon = 1.000; heterozygotes vs all homozygotes: OR = 0.854, 95%CI = 0.678–1.077, *P* = 0.183, Bon = 1.000). Between-study heterogeneity was not detected in all the contrasts. The association between the PPARγ Pro12Ala polymorphism and CD in the European Caucasian population was investigated in 3 studies with 979 CD cases and 1387 controls [10], [20], [21]. The AlaAla genotype seemed to protect the European Caucasian population against the development of CD in a recessive way(Pro vs Ala: OR = 1.135, 95%CI = 0.951–1.354, *P* = 0.162, Bon = 1.000; ProPro vs ProAla: OR = 1.042, 95%CI = 0.852–1.273, *P* = 0.690, Bon = 1.000; ProPro vs AlaAla: OR = 2.379, 95%CI = 1.110–5.100, *P* = 0.026, Bon = 0.156; ProAla vs AlaAla: OR = 2.315, 95%CI = 1.064–5.037, *P* = 0.034, Bon = 0.204; Pro homozygotes vs Ala positives: OR = 1.094, 95%CI = 0.899–1.330, *P* = 0.371, Bon = 1.000; Pro positives vs Ala homozygotes: OR = 2.360, 95%CI = 1.103–5.053, *P* = 0.027, Bon = 0.162; heterozygotes vs all homozygotes: OR = 0.976, 95%CI = 0.799–1.192, *P* = 0.809, Bon = 1.000). Between-study heterogeneity was not detected in all the contrasts.

### Analysis in the East Asian population

The association between the PPARγ Pro12Ala polymorphism and UC in the East Asian population was investigated in 3 studies with 359 UC cases and 496 controls [9], [11], [19]. There was no significant association between this polymorphism and UC in the East Asian population (Pro vs Ala: OR = 0.576, 95%CI = 0.180–1.844, *P* = 0.353, Bon = 1.000; ProPro vs ProAla: OR = 0.856, 95%CI = 0.502–1.460, *P* = 0.568, Bon = 1.000; ProPro vs AlaAla: OR = 0.490, 95%CI = 0.089–2.700, *P* = 0.413, Bon = 1.000; ProAla vs AlaAla: OR = 0.774, 95%CI = 0.128–4.669, *P* = 0.780, Bon = 1.000; Pro homozygotes vs Ala positives: OR = 0.686, 95%CI = 0.284–1.654, *P* = 0.401, Bon = 1.000; Pro positives vs Ala homozygotes: OR = 0.500, 95%CI = 0.091–2.750, *P* = 0.425, Bon = 1.000; heterozygotes vs all homozygotes: OR = 1.164, 95%CI = 0.683–1.984, *P* = 0.577, Bon = 1.000). Between-study heterogeneity was detected in the contrasts of Pro vs Ala and Pro homozygotes vs Ala positives. The association between the PPARγ Pro12Ala polymorphism and CD in the East Asian population was investigated in 2 studies with 42 CD cases and 354 controls [9], [11].There was no significant association between this polymorphism and CD in the East Asian population (Pro vs Ala: OR = 1.086, 95%CI = 0.349–3.382, *P* = 0.886, Bon = 1.000; ProPro vs ProAla: OR = 0.933, 95%CI = 0.289–3.012, *P* = 0.907, Bon = 1.000; ProPro vs AlaAla: OR = 0.324, 95%CI = 0.029–3.663, *P* = 0.363, Bon = 1.000; ProAla vs AlaAla: OR = 0.458, 95%CI = 0.031–6.825, *P* = 0.571, Bon = 1.000; Pro homozygotes vs Ala positives: OR = 1.011, 95%CI = 0.315–3.249, *P* = 0.985, Bon = 1.000; Pro positives vs Ala homozygotes: OR = 0.321, 95%CI = 0.028–3.623, *P* = 0.358, Bon = 1.000; heterozygotes vs all homozygotes: OR = 1.082, 95%CI = 0.335–3.493, *P* = 0.896, Bon = 1.000). Between-study heterogeneity was not detected in all the contrasts.

### Analysis in the Turkish population

The association between the PPARγ Pro12Ala polymorphism and IBD in the Turkish population was investigated in 1 study with 45 UC cases, 69 CD cases and 100 controls [18]. And this study showed no significant association between this polymorphism and UC or CD in the Turkish population.

Moreover, the conclusions of our meta-analysis did not change when we used a fixed model and a random model to perform our meta-analysis respectively.

### Evaluation of reporting bias

**Figure 4:**
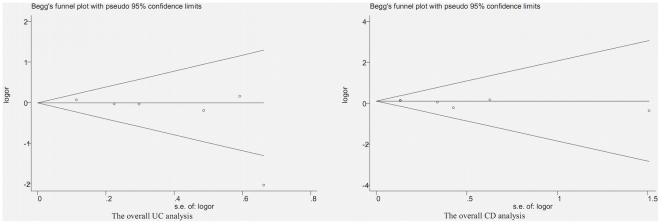
Begg funnel plots of the overall UC analysis and the overall CD analysis.

Obvious asymmetry was revealed by the shapes of the Begg funnel plots in the overall UC analysis and the overall CD analysis. The study with the smallest sample size reported by Aoyagi was a clear outlier which might be suggestive of a small-study effect. The Begg funnel plots corresponding to the allelic analysis are shown in Figure 4.

## Discussion

The core pathophysiological process of UC is the uncontrolled inflammation mainly affecting the colon. Environmental factors including commensal flora and other antigens and host factors including genetic dispositions are all involved in the UC development [3], [4]. The primarily upregulated response of the intestinal mucosal immune system is probably responsible for the uncontrolled inflammation of UC [4].

Firstly, dysfunction of the cellular and immune intestinal barrier which separates the microbial flora from host tissues renders a host susceptible to invasive factors [22]. Secondly, environmental factors including the dietary factor and microbia activate the Toll-like receptors (TLRs), which then activate NF-κB and cause the expression of pro-inflammatory genes consequently [23]. In the inflammation regulation networks, activation of PPARγ could suppress the activation of NFκB and TLRs [24], [25], therefore inhibit the cascades of inflammation. Decreased PPARγ expression in epithelial cells was also found in UC patients in one study [26]. Moreover, a randomized placebo-controlled trial suggested that a PPARγ agonist was efficacious in the treatment of mild to moderately active UC [27]. All of these indicate normal expression of PPARγ as a protecting factor against the development of UC. Pro12Ala mutation is a common mutation, which downregulates the activity of PPARγ as indicated in one study [28]. Therefore the Pro12Ala mutation may be a risk factor for UC. However, our meta-analysis did not find a significant association between this mutation and the development of UC in the overall, European Caucasian and East Asian population. Our explanation is that the Pro12Ala mutation only affects the function of PPARγ2, and the function of PPARγ1 which makes up a large portion of PPARγ in intestines is intact. So the effect of the Pro12Ala mutation on the whole PPARγ function is limited, which would attenuate the effect of the Pro12Ala mutation on UC.

The mechanism of the uncontrolled inflammation in CD is different from that in UC. The deficiency of the innate immune system which permits intestinal pathogens to break through the intestinal barrier and activates the adaptive immune system may be responsible for the uncontrolled inflammation in CD [4]. The impaired function of macrophages may be the central process in the innate immune system deficiency [4], [29], [30], which causes granulomas in CD consequently. Nod proteins and PPARγ are all involved in the maintenance of normal innate immune system [6], [31]. Gene mutations of the elements constituting the innate immune system may contribute to the CD development. Genetics play more important roles in the development of CD than in the development of UC [32], [33]. NOD2/CARD15 polymorphism is the first confirmed risk factor for CD in Caucasian populations [34]. This encourages researchers to find other CD susceptible gene polymorphisms. A recent meta-analysis of genome-wide association scans identified *PTPN2, IL18RAP, TAGAP*, and *PUS10* loci as new CD risk factors [35], which widened our understanding of the gene background of CD. The PPARγ Pro12Ala mutation would decrease the function of PPARγ [28], and consequently abate its suppression on the activation of NFκB and TLRs [24], [25], which would enhance the function of the innate immune system and protect a host against the development of CD.

Our meta-analysis supports this hypothesis. The AlaAla genotype seemed to protect the European Caucasian population against the development of CD. The Pro12Ala mutation only affects the function of PPARγ2, which supports PPARγ2 as a modulator in the innate immune system maintenance. However, we did not find an association between this polymorphism and CD in the East Asian population. This is in consistence with the observation of NOD2/CARD15 polymorphism on CD in the non Caucasian populations [36], [37]. We deduce that the PPARγ Pro12Ala mutation, the NOD2/CARD15 polymorphism and other unknown gene polymorphisms mainly occurred in the Caucasian population may function synergistically in the CD development. Further these polymorphisms may constitute the gene background of the Caucasian population in turn.

However, the conclusions of this meta-analysis should be interpreted cautiously. This meta-analysis has some limitations. Firstly, only one study used colonoscopy to exclude IBD in the controls [9]. Only one study described the severity of cases, which was mild to moderate [20]. All of the above could cause selection bias in some studies. Secondly, only two studies described the measures for genotyping quality control [11], [21], and two studies used blindness measures [19], [21]. Information bias could not be excluded completely. Thirdly, the controls were slightly older as compared to CD patients in one study [20], which would affect the comparability between arms in this study. Fourthly, the number of included studies was not large enough although we endeavored to collect relevant full text published studies. Fifthly, Obvious asymmetry was revealed by the shapes of the Begg funnel plots in the overall UC analysis and the overall CD analysis. The study with the smallest sample size reported by Aoyagi was a clear outlier, which might be suggestive of a small-study effect. Thus a reporting bias cannot be excluded. More high quality and large sample studies evaluating the association between the Pro12Ala mutation and the presence of IBD are still needed. An update of our meta-analysis is necessary in the future. Furthermore, only one study evaluated the interaction between diets and the Pro12Ala polymorphism which showed that a high intake of saturated and mono-unsaturated fat was associated with a more active CD only in wild type carriers [20]. The combined effect of diets and this polymorphism still needs to be elucidated. Studies investigating the interaction between the PPARγ Pro12Ala mutation and the NOD2/CARD15 polymorphism in the CD development in the Caucasian population are also wanted.

In conclusion, based on published full text case-control studies, our meta-analysis demonstrates that the AlaAla genotype may be a protecting factor in the European Caucasian population against the development of CD in a recessive way.

## Supporting Information

Text S1 Checklist
(DOC)

Text S2 Scopus searching result
(DOC)
