# Supplementary material for: Association between the Pro12Ala Polymorphism of Peroxisome Proliferator-Activated Receptor Gamma 2 and Inflammatory Bowel Disease: A Meta-Analysis
Source: PLoS One. 2012 Jan 19;7(1):e30551. doi: 10.1371/journal.pone.0030551 (PMC3261897; doi:10.1371/journal.pone.0030551)
Supplement: Text S2 — Scopus searching result (DOC) [file pone.0030551.s002.doc]

Scopus searching

1. TITLE-ABS-KEY(inflammatory bowel disease) 30,917
2. TITLE-ABS-KEY(crohn's disease) 30,415
3. TITLE-ABS-KEY(crohn disease) 47,819
4. TITLE-ABS-KEY(ulcerative colitis) 41,177
5. (TITLE-ABS-KEY(colitis) AND NOT TITLE-ABS-KEY(infectious) 65,580
6. (TITLE-ABS-KEY(colitis) AND NOT TITLE-ABS-KEY(ischemic) 64,469
7. TITLE-ABS-KEY(ibd) 10,472
8. TITLE-ABS-KEY(uc) 13,003
9. TITLE-ABS-KEY(cd) 213,656
10. #1 OR #2 OR #3 OR #4 OR #5 AND #6 OR #7 OR #8 OR #9 320,977
11. TITLE-ABS-KEY(peroxisome proliferator-activated receptor) 22,387
12. TITLE-ABS-KEY(ppar) 15,291
13. #11 AND #12 23,820
14. TITLE-ABS-KEY(polymorphisms) 270,572
15. TITLE-ABS-KEY(polymorphism) 270,572
16. TITLE-ABS-KEY(single nucleotide) 128,040
17. TITLE-ABS-KEY(allele) 194,191
18. TITLE-ABS-KEY(alleles) 194,191
19. TITLE-ABS-KEY(genotype) 259,387
20. TITLE-ABS-KEY(genotypes) 259,387
21. #14 OR #15 OR #16 OR #17 OR #18 OR #19 OR #20 578,115
22. #10 AND #13 AND #21 56
